# Fractional Flow Reserve Assessment of a Significant Coronary Stenosis Masked by a Downstream Serial Lesion

**DOI:** 10.1155/2016/1987238

**Published:** 2016-07-27

**Authors:** Lawrence Yu-Min Liu, Hsu-Ping Wu, Po-Lin Lin

**Affiliations:** Division of Cardiology, Department of Internal Medicine, Mackay Memorial Hospital Hsinchu, No. 690 Section 2, Guangfu Road, Hsinchu 30071, Taiwan

## Abstract

Fractional flow reserve (FFR) has been recognized as an effective tool to determine functional significance in intermediate coronary lesions and FFR-guided percutaneous coronary intervention (PCI) improves clinical outcomes. However, hemodynamic interaction between serial stenoses within one coronary artery complicates the assessment of functional severity of each individual lesion. We present a case in which FFR measurement by intracoronary bolus injection of adenosine helps to make appropriate revascularization decision in serial stenoses when the procedures are performed systemically and properly.

## 1. Introduction

Fractional flow reserve (FFR) has been recognized as an effective tool to determine functional significance in intermediate coronary lesions. FFR is defined as the ratio between mean distal coronary pressure and mean aortic pressure at maximal hyperemia. A 0.014-inch wire with pressure sensor tip is introduced and advanced across the target lesions. The FFR value is measured after continuous intravenous infusion or intracoronary bolus injection of adenosine to induce maximal coronary artery vasodilatation. Several landmark outcome trials showed that FFR-guided PCI significantly reduced major adverse cardiac events including death and urgent revascularization in patients with stable coronary artery disease [1, 2]. Functionally insignificant lesions can also be safely treated with optimal medical therapy and unnecessary PCI might be avoided. Current practice guidelines recommend utilization of FFR to identify functionally significant lesions in intermediate lesions and a cutoff of FFR ≤ 0.8 is used to guide coronary revascularization [3, 4]. Nevertheless, identifying which lesions are functionally significant is more challenging in vessels with serial or multiple stenoses. Understanding of the basic physiology and knowledge of FFR methods are important to the correct interpretation of the measurements, especially in complex lesions. We herein present a case in which FFR helps to guide appropriate PCI treatment in a single coronary artery with serial stenoses.

## 2. Case Presentation

**Figure 1:**
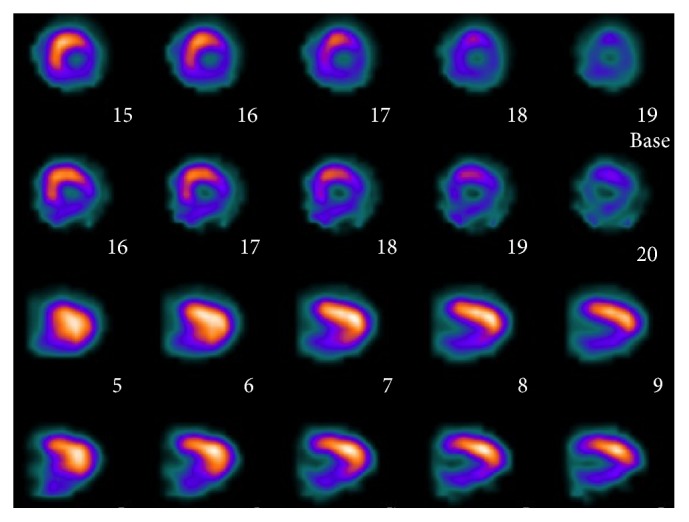
Myocardial perfusion scan showed myocardial ischemia at the lateral and inferior segments of left ventricle.

**Figure 2:**
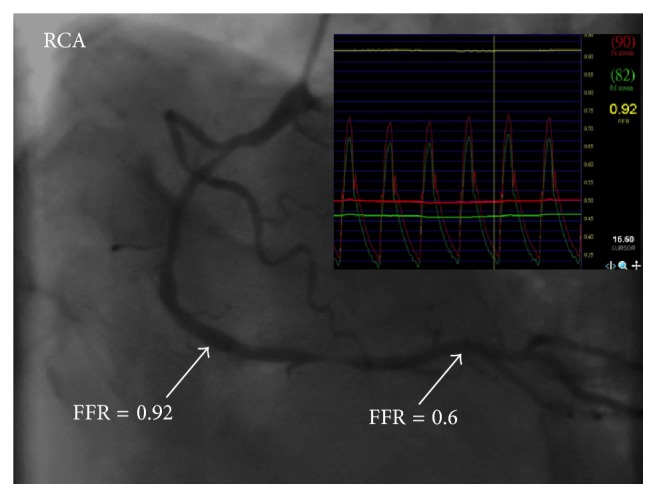
Angiography revealed a proximal 60% stenosis and a distal 90% stenosis in the right coronary artery.

**Figure 3:**
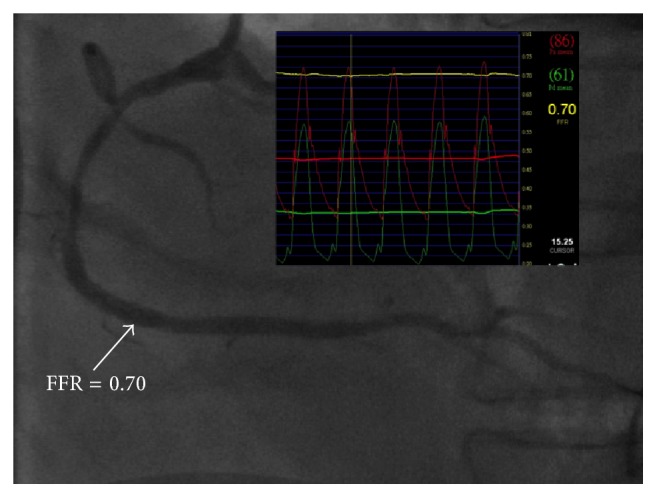
FFR decreased from 0.92 to 0.7 distal to the proximal stenosis after a drug-eluting stent was deployed in the distal right coronary artery.

**Figure 4:**
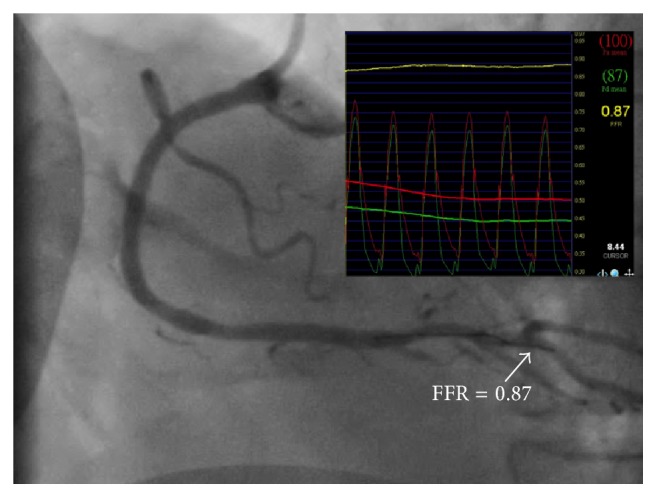
Final angiography and FFR after the serial stenoses in the right coronary artery were both stented.

A 67-year-old male with hypertension, type 2 diabetes, and hyperlipidemia had a 2-month history of effort angina (Canadian Cardiovascular Society class 3). Echocardiography showed left ventricular hypertrophy and normal systolic function with ejection fraction of 67%. Myocardial perfusion scan revealed a large area of myocardial ischemia at the lateral and inferior segments of left ventricle (Figure 1). Invasive coronary angiography demonstrated a critical stenosis in the mid left circumflex artery, which was stented with a drug-eluting stent. There were also an intermediate coronary lesion in the proximal right coronary artery (RCA) and a very severe stenosis 40 mm downstream of the proximal lesion. A 0.014-inch FFR pressure wire (PressureWire, St. Jude Medical, Uppsala, Sweden) was advanced beyond both lesions and intracoronary boluses of adenosine were given to achieve maximal hyperemia. FFR was 0.6 after administration of 48 *μ*g of intracoronary adenosine and FFR became 0.92 after 96 *μ*g of intracoronary adenosine when the pressure sensor was pulled back and positioned between the two lesions (Figure 2). Percutaneous coronary intervention (PCI) using a drug-eluting stent for the distal stenosis was performed. Repeat FFR after PCI measured precisely at the previous position between the two lesions decreased significantly to 0.7 after 48 *μ*g of intracoronary adenosine (Figure 3). PCI using a drug-eluting stent for the proximal intermediate lesion was therefore performed. Subsequent FFR in the distal RCA was 0.87 after both lesions were treated (Figure 4). The patient was discharged the following day and free of symptoms after one year.

## 3. Discussion

This case demonstrates that measurement of FFR in an intermediate lesion is critical in guiding PCI but can be easily misinterpreted in the presence of a serial lesion in the same artery.

When serial or multiple stenoses are present in the same coronary artery, hemodynamic interaction between stenoses complicates the assessment of functional severity of individual lesions. FFR value of each stenosis is usually underestimated and this influence is more pronounced for the proximal lesion than for the distal one. Individual FFR of each stenosis can be predicted by the following equation: FFR predicted = (*P*_*d*_ − [(*P*_*m*_/*P*_*a*_)*∗P*_*w*_])/((*P*_*a*_ − *P*_*m*_)+(*P*_*d*_ − *P*_*w*_)) [5]. The equation uses arterial pressure (*P*_*a*_), pressure between the two stenoses (*P*_*m*_), distal pressure (*P*_*d*_), and coronary occlusion wedge pressure (*P*_*w*_) during maximum hyperemia. The higher coronary wedge pressure created by a more severe distal lesion results in an even more pronounced underestimation for the proximal lesion as our case evidently displayed. Nonetheless, FFR remains a helpful tool to improve PCI outcomes and reduce unnecessary intervention in these complex lesions. Kim et al. studied a total of 131 patients with multiple intermediate stenoses and 182 out of the 298 lesions were deferred according to FFR measurements. There were no events related to the deferred lesion at follow-up [6].

Most of the FFR studies were performed with a continuous intravenous administration of adenosine at a rate 140 *μ*g/kg/min [7]. Previous reports on serial stenoses within one coronary artery also advocated intravenous infusion of adenosine and pullback pressure tracing to induce maximal hyperemia and to identify functionally significant lesions. This approach is especially effective to recognize all significant pressure gradients in serial or diffuse lesions. However, it is paramount to repeat FFR measurement after treatment of the first targeted lesion to avoid missing other significant lesions that might be influenced or underestimated during the first measurement. Although we utilized a different approach using intracoronary adenosine to achieve maximal hyperemia, we followed the same measurement principle by performing repeat FFR after each treatment, which also yielded excellent angiographic and FFR results.

Despite the recommended use of intravenous route of adenosine administration, intracoronary adenosine is still frequently used in clinical practice and published data [8]. Intracoronary adenosine is found to be associated with reduced procedural time and improved patient comfort at a lower cost without sacrificing FFR accuracy and patient safety [9]. In our case, a relatively lower dose of intracoronary adenosine was started to induce maximal hyperemia. If FFR was greater than 0.8 at the initial dose, we increased the dose of adenosine until FFR value did not change with the higher dose. De Luca et al. showed that high doses of intracoronary adenosine increased the sensitivity of FFR [10]. Another study further specified an intracoronary adenosine bolus injection of 100 *μ*g in the RCA and 200 *μ*g in the LCA induced maximum hyperemia while being associated with minimal side effects [11].

A recent development in functional measurement of coronary stenosis using a hyperemia-free, pressure-derived hemodynamic index can potentially mitigate problems associated with hyperemic FFR in serial lesions. Instant wave-free ratio (iFR) is a resting index calculated as the ratio of distal coronary pressure to proximal aortic pressure over a specific wave-free period in diastole. During this cardiac cycle, resistance is naturally constant and minimized without administration of vasodilator drugs. This resting index measured at basal state can eliminate the flow interaction between stenoses under hyperemia. An iFR-pullback method can create a physiological map in complex serial stenoses and diffuse vessels and predict an expected iFR value for the treatment of selected stenosis [12, 13]. Moreover, using a hybrid iFR-FFR strategy can free 65.1% of patients and 69.1% of stenoses from hyperemia and greatly simplify functional assessment during PCI [14].

This case highlighted the importance of FFR in evaluating intermediate lesion and how it could be easily misinterpreted if operators are not familiar with this diagnostic tool. It also demonstrated that FFR measurement using intracoronary adenosine in serial lesions is a feasible and timesaving approach if correct and necessary procedures are performed systemically.
